# How do orthopaedic surgeons inform their patients before knee arthroplasty surgery? A cross-sectional study

**DOI:** 10.1186/s12891-018-2345-7

**Published:** 2018-11-24

**Authors:** Aamir Mahdi, Maria Hälleberg Nyman, Per Wretenberg

**Affiliations:** 1 Department of Orthopaedics, Örebro County, Sweden; 2 Faculty of Medicine and Health, School of Medical Sciences, Örebro University, Örebro, Sweden; 3 Faculty of Medicine and Health, School of Health Sciences, Örebro University, Örebro, Sweden

**Keywords:** Expectation, Postoperative outcome, Preoperative information, Psychiatric history, TKA

## Abstract

**Background:**

Total knee arthroplasty (TKA) is a successful and common procedure. However, 6–28% of patients are dissatisfied postoperatively. The provision of preoperative patient information, inquiring about patients’ expectations, and taking a psychiatric history are essential parts of both preoperative evaluation and postoperative outcome. The aim of this study was to investigate how orthopaedic knee surgeons in Sweden inform their patients before surgery.

**Methods:**

A questionnaire was distributed to all knee surgeons performing TKA in Sweden. Responses were received from 60 of the 65 orthopaedic departments performing TKA in Sweden (92%), covering 219 of the approximately 311 knee surgeons at the 65 departments (70%). The answers were analysed with descriptive statistics. A content analysis of the surgeons’ opinions was also performed using a thematic method.

**Results:**

In terms of information provision, 58% of the surgeons always gave written information while 92% informed orally. Only 44% always asked about the patient’s expectations, and only 42% always informed patients about the 20% dissatisfaction rate after TKA. Additionally, 24% never operated on mild indication of arthrosis, 20% always took a psychiatric history, and half never or seldom consulted a psychiatrist. However, all the knee surgeons believed in a psychiatric impact on TKA outcome. Qualitative analysis revealed five common causes of patient dissatisfaction, which in descending frequency were: patients’ expectations, choice of patients to operate on, surgical factors, combinations of factors, and insufficient information provision to patients.

**Conclusions:**

Knee surgeons in Sweden have considerable awareness of the importance of preoperative patient information, the impact of patient expectations, and psychiatric illness. However, they need to improve their preoperative routines when it comes to providing written information, asking about the patient’s expectations, and psychiatric assessment.

## Background

Total knee arthroplasty (TKA) is a common procedure with generally good results. By 2030, the annual number of TKA operations performed in the USA is expected to have reached 3.48 million, an increase of 673% from the figures for 2005 [1]. The most recent annual report from the Swedish knee arthroplasty register shows a similar trend, with an increase from 70 to 140 TKA procedures per 100,000 inhabitants between 2000 and 2015 [2]. However, despite advances in design and technique, there are still patients who are not satisfied after this procedure. Previous studies have shown many reasons for dissatisfaction [3–24], for example early- and late postoperative complications, unfulfilled expectations, anxiety and depression. Further, earlier studies have shown a correlation between dissatisfaction and mechanical [5, 14] and/or psychological factors [16]. In Sweden, the risk of dissatisfaction after knee arthroplasty is about 8% in the absence of complications [22, 23].

Patient dissatisfaction contradicts the aim of TKA surgery in improving patient’s quality of life [10, 25–27]. It implies a burden for both patients and health care professionals [28–32]. Most of the previous studies conducted to address the problem of dissatisfaction and to analyse the preventable factors in order to decrease this rate. [6, 10, 12, 15, 25, 26, 33–38]

Orthopaedic surgeons may have different ways of dealing with how patients are informed preoperatively, as well as different opinions about the importance of preoperative information in relation to postoperative outcome. Preoperative information includes a general written and verbal information, patient expectations, information about the 20%-non-satisfaction’s rate and psychiatric history. A previous study showed a discordance between surgeon and patient satisfaction after knee arthroplasty surgery [39]. Moreover, a qualitative study by Conrades et al. showed a relationship between how the TKA patient was informed preoperatively and how much they trusted the department [40].

When it comes to reducing the risk for patient dissatisfaction, we know the importance of a qualified preoperative information according to the patient’s needs and selection of the right patient for surgery [12, 18, 40–44], but we do not know the extent of the problem in Sweden in terms of the surgeon’s attitude to the information, patient selection, and the surgeon’s opinion about patient dissatisfaction. There are few studies which describe orthopaedic surgeons’ attitudes toward giving information to patients. The aim of this study was therefore to investigate how TKA surgeons in Sweden inform their patients preoperatively, and what kind of information they give. This will lead to strategies and possibly changing protocols to improve the knee surgeon’s attitude in preoperative patient’s information. In turn, it will possibly decrease the dissatisfactions rate in Sweden.

## Methods

### Design

We conducted a cross-sectional descriptive study including qualitative and quantitative data collection.

### Methods and analyses

A study-specific questionnaire was distributed in paper form to all 65 TKA clinics in Sweden in May 2016. A list of all orthopaedic clinics performing TKA was obtained from the Swedish knee registry. A reminder letter was sent in October 2016 to increase the response rate. For each department, the questionnaire was sent to the orthopaedic surgeon locally responsible for registration of TKAs in the Swedish Knee Arthroplasty Register, who then distributed the questionnaire to all orthopaedic surgeons who performed TKA at that department. Knee departments in Sweden are located in either state-funded university hospitals, regional hospitals, small hospitals, or private clinics.

The initial response rate was 55%, but the reminder letter increased this to 92% (60 clinics) (Appendix 1). The five orthopaedic clinics that failed to return the questionnaire were located in a university hospital (*n* = 1) and local hospitals (*n* = 4). Of the approximately 311 knee arthroplasty surgeons in Sweden, 219 answered the questionnaire. Thus, about 70% of the surgeons who regularly perform TKA surgery in Sweden answered the questionnaire. The reasons of 30% non-response were the surgeon’s unwilling and uninterestingness according to orthopaedic chief managers. This rate was not related to the kind of hospital (University hospital, county council hospital or local hospital).

The sixteen-item questionnaire was designed by the researchers with the aim of investigating how information was given to and discussed with patients before surgery. The first fourteen questions covered written and oral information, patients’ expectations, surgery on mild indication, and patients’ psychiatric history. The choice of questions was based on evidence from the literature that these factors can have an impact on outcome [4, 9, 12, 13, 16, 19, 20, 32, 42–45]. A five-point Likert scale was used, with response alternatives of always, often, sometimes, seldom, and never after a discussion with a statistician (Appendix 2).

The final two questions were concerned with what the surgeons believed were the most common reasons for dissatisfaction after TKA, and were answered in the form of free text. These two questions were analysed qualitatively with a quantitative component. The text was coded inductively and grouped into five main categories based on 262 responses, and then analysed with a method based on thematic qualitative analysis [46, 47]. Initial coding was performed by the first author(AM). Then, the second author(MH) checked the quality of the coding and changes were made until consensus was reached. To facilitate thematic analysis, responses on the two-free text items were uploaded into Version 11 of the NVivo software package (Boston, MA, USA).

There was no previous validated questionnaire which cover all the items that we intended to study. The questionnaire was written in Swedish by the major supervisor and the main researcher. The co-supervisor made then several changes on the language and the construct. It sent then to two independent orthopaedic surgeons to study the construct of the questionnaire. Further changes were made to the final version of the questionnaire. No comments have been received from the ethical board committee or knee orthopaedic surgeons if something in the questionnaire were ambiguous or not-understandable.

#### Statistical analysis

The statistical data analysis was divided into two parts:

1. **General analysis:** The data were described in terms of frequencies of answers. Duration of experience was not taken into consideration.
2. **Specific analysis:** The surgeons were divided into subgroups classified in terms of years of experience, volume of primary TKA/revision surgeries per year, and percentage of work done on knees. For statistical purposes, orthopaedic surgeons who performed 75% of their work on knees were grouped with those working solely on knees. Subgroup analysis was performed to see if there were differences between the groups (Table 1).

**Table 1:** Characteristics of Swedish knee surgeons (*N* = 219)

Variable	Orthopaedic knee surgeon (*N* = 219)
Experience, *n* (%)	
< 5 years	32 (15)
5–15 years	95 (43)
15–30 years	80 (37)
> 30 years	11 (5)
Missing	1 (0.5)
^a^Knee specialist, *n* (%)	
100% Knee specialist	18 (8)
50% knee specialist	81 (37)
25% Knee specialist	120 (55)
Primary TKA/year, *n* (%)	
< 22	33 (15)
22–50	103 (47)
50–100	54 (25)
> 100	24 (11)
Missing	5 (2)
Revision TKA/year, *n* (%)	
No revision	98 (45)
< 5 revisions	54 (25)
> 5 revisions	65 (29)
Missing	2 (1)

The table shows the classification of knee surgeons according to the years of experience, the percentages of daily clinical work with knee joint, the volume of primary TKA performed by every surgeon per year and the volume of revision knee replacement surgery performed by every surgeon per year

^a^Percentage of daily clinical work with knee joint

Data were analysed as frequencies of each answer (always, often, sometimes, seldom, and never). More specifically, subgroup data analysis was performed to see if there were any statistically significant differences (Table 1). The chi-square test was used to compare multiple categorical groups. Version 24 of the SPSS software package (IBM, SPSS Inc., Chicago, Illinois, USA) was used for the quantitative data analysis.

## Results

### Descriptive statistics

Only 8% of orthopaedic surgeons in Sweden worked solely on TKA surgery. In terms of experience, 43% had 5–15 years of experience in knee arthroplasty, 47% performed 22–50 primary TKA procedures per year, and more than 50% performed revision arthroplasty of varying degrees of complexity (Table 1). The results are presented below in terms of the nine main questions. Those represented question 1,2,7,8,9,10,11,13 and 14. The answers of question 12 were presented together with question 13. Question 3,4,5 and 6 were dealt with knee surgeons experiences (Table 1, Appendix 2). The answer of question 15 and 16 were analysed qualitatively (Appendix 2).

#### Written information

The results showed that 58% of the knee surgeons always gave written information to their patients, while 18% never gave written information (Table 2). The highest percentages of written information were given by surgeons with more than 30 years of experience (64%), who performed more than 100 primary TKA procedures per year (71%), who performed more than 5 revisions per year (60%), and who worked solely on knees (67%). However, there was no statistically significant differences between the groups (Table 3).

**Table 2:** Knee surgeons’ measures regarding preoperative patient information

Variable	Orthopaedic knee surgeon (*N* = 219)
Written information, *n* (%)	
Always	127 (58)
Often	24 (11)
Sometimes	7 (3.2)
Seldom	19 (9)
Never	40 (18)
Oral information, *n* (%)	
Always	202 (92)
Often	16 (7)
Sometimes	1 (1)
Seldom	0 (0)
Never	0 (0)
Patient expectation, *n* (%)	
Always	97 (44)
Often	78 (36)
Sometimes	32 (15)
Seldom	9 (4)
Never	3 (1)
Dissatisfaction rate, *n* (%)	
Always	91 (42)
Often	76 (35)
Sometimes	27 (12)
Seldom	15 (7)
Never	9 (4)
Mild indications, *n* (%)	
Always	2 (1)
Often	12 (6)
Sometimes	54 (25)
Seldom	98 (45)
Never	52 (24)
Psychiatric consultation, *n* (%)	
Always	21 (10)
Often	38 (17)
Sometimes	45 (21)
Seldom	75 (35)
Never	38 (17)
Impact of psychiatric disorder, *n* (%)	
Always	63 (29)
Often	119 (54)
Sometimes	34 (16)
Seldom	3 (1)
Never	0 (0)
Psychiatric history, *n* (%)	
Always	42 (19)
Often	83 (38)
Sometimes	59 (27)
Seldom	28 (13)
Never	7 (3)
Psychiatric questionnaire, *n* (%)	
No	213 (97)
Yes	2 (1)
EQ-5D	3 (1)
HADS	1 (1)

The table shows the numbers and percentages of all knee surgeons regarding the given preoperative patient’s information

**Table 3:** The sub groups of knee surgeons who gave the highest preoperative information (*N* = 219)

Characteristics of knee surgeons:	Years of experience	*P*-value*	TKA volume per year	*P*-value*	Knee revision per year	*P*-value*	Knee specialist	*P*-value*
Preoperative measures:								
Written information	> 30	0.72	> 100	0.25	> 5	0.31	100%	0.24
Oral information	15–30	0.39	22–50	0.50	0	0.33	50%	0.32
Expectations	> 30	0.22	> 100	0.51	0	0.19	50%	0.08
Dissatisfaction rate	> 30	0.69	> 100	0.17	< 5	0.47	50%	0.79
Surgery on mild indication	> 30	0.89	< 22	0.70	< 5	0.04	25%	0.77
Psychiatric history	< 5	0.09	> 100	0.59	< 5	0.70	50%	0.94
Psychiatric consultation	> 30	0.03	22–50	0.07	0	0.31	25%	0.07
Psychiatric impact	< 5	0.14	22–50	0.36	0	0.18	50%	0.38

The table shows the highest preoperative given information according to different groups of knee surgeons that were described in Table 1

*Chi ^2^ test

Surgeons who said they never gave written information had the following characteristics: 15–30 years of experience (23%), performed fewer than 22 primary TKA procedures per year (27%), did not perform any knee revisions (21%), and spent 25% of their time as knee surgeons (23%) (Table 4).

**Table 4:** The sub groups of knee surgeons who gave the least preoperative information (*N* = 219)

Characteristics of knee surgeons:	Years of experience	*P*-value*	TKA volume per year	*P*-value*	Knee revisions per year	*P*-value*	Knee specialist	*P*-value*
Preoperative measures:								
Written information	15–30	0.72	< 22	0.25	0	0.31	25%	0.24
Oral information	< 5	0.39	50–100	0.50	< 5	0.33	50%	0.32
Expectations	< 5	0.22	50–100	0.51	< 5	0.19	100%	0.08
Dissatisfaction rate	< 5	0.69	> 100	0.17	0	0.47	100%	0.79
Surgery on mild indication	< 5	0.89	50–100	0.70	< 5	0.04	50%	0.77
Psychiatric history	> 30	0.09	50–100	0.59	> 5	0.70	100%	0.94
Psychiatric consultation	< 5	0.03	50–100	0.07	0	0.31	100%	0.07
Psychiatric impact	15–30	0.14	> 100	0.36	> 5	0.18	50%	0.38

This table shows the lowest preoperative given information according to different groups of knee surgeons that were described in Table 1

*Chi ^2^ -test

#### Oral information

Almost all knee surgeons informed their patients orally about the procedure. Oral information included indications, contraindications of surgery, benefits, risks, expected results, and prognosis. In terms of consistency, 92% always informed their patients, 7% often informed their patients, and 1% sometimes informed their patients (Table 2).

Further classification of surgeons into subgroups did not show any statistically significant differences. The highest proportions of surgeons informing their patients orally were seen in surgeons with 15–30 years of experience (94%), performing 22–50 primary TKA procedures per year (95%), performing no revisions (94%), and conducting 50% of their work on knees (95%) (Table 3).

While experienced knee surgeons did not report the highest frequency of providing oral information, the percentage who did provide oral information was still high and there were no statistically significant differences between the groups. The lowest frequencies of giving oral information were in surgeons who “sometimes” informed patients verbally as follows: < 5 years of experience (3%), 50–100 primary TKA procedures per year (2%), < 5 revisions per year (1%), and conducting 50% of their work on knees (1%) (Table 4).

#### Expectations

There was an unequal distribution of orthopaedic surgeons asking about the patient’s expectations: 44% always asked, 35% often asked, 14% sometimes asked, and the remaining 5% never or seldom asked (Table 2).

In terms of subgroups, 82% of surgeons with more than 30 years of experience, 54% of surgeons who performed more than 100 primary TKA procedures per year, 47% of surgeons who did not perform revisions, and 47% of surgeons who conducted 50% of their work on knees “always” asked about patient expectations. However, the differences again were not statistically significant (Table 3). The lowest results were among the following, who “never” asked about expectations: < 5 years of experience (3%), 50–100 TKA procedures per year (4%), < 5 revisions per year (4%), and specialising 100% in knees (6%) (Table 4).

#### Informing patients about the 20% dissatisfaction rate after TKA surgery

Only 42% of surgeons always informed patients about the risk of dissatisfaction; 35% often provided this information while approximately 10% never or seldom informed patients (Table 2). Surgeons who informed patients frequently had the following characteristics: > 30 years of experience (37%), > 100 primary TKA procedures per year, < 5 knee revisions per year (52%), and conducting 50% of their work on knees (49%) (Table 3). The lowest results were found among the following categories: < 5 years of experience (6%), > 100 primary TKA procedures per year (8%), no knee revisions (6%), and specialising 100% in knees (6%) (Table 4).

#### Surgery on mild indications

Only 24% of the surgeons never operated on mild indication, though an additional 45% seldom proceeded with surgery in these cases. However, the proportion of surgeons who sometimes, often, or always operated despite a suspicious indication was still high, at 31% (Table 2).

The most appropriate answer on this issue is “seldom” [30, 45]. Orthopaedic surgeons who provided those answers had the following characteristics: > 30 years of experience (55%), < 22 TKA procedures per year (55%), < 5 revisions per year (57%, *p* = 0.04), and conducting 25% of work on knees (45%) (Table 3). Surgeons who gave the answer of “always” operating on mild indication had the following characteristics: < 5 years of experience, 50–100 primary TKA procedures per year (4%), < 5 revisions per year (2%) and conducting 50% of their work on knees (1%) (Table 4).

#### Psychiatric consultation

Despite the known impact of psychiatric illness on TKA outcome, only one in ten of the surgeons always consulted a psychiatric unit; 17% did this often and a fifth did it sometimes (Table 2).

Stratifying the surgeons into four groups showed that surgeons who mostly answer “always” had the following criteria: > 30 years of experience (18%, *p* = 0.03), 22–50 primary TKA procedures per year (15%), no revisions (12%), and spending 25% of their time on knees (13%). The only subgroup with a statistically significant difference was the group with more than 30 years of experience. Knee surgeons who never consulted a psychiatrist had the following characteristics: < 5 years of experience (25%), 50–100 primary TKA procedures per year (22%), no revisions (24%), and specialising 100% in knees (Table 4).

#### Impact of psychiatric problems on outcome

A total of 29% of the surgeons believed that psychiatric problems always had a negative impact on outcome after TKA, 54% believed there was often an impact, and 16% believed there was sometimes an impact. Although 2% answered “rarely” to this question, there were no surgeons who answered “never” (Table 2).

The knee surgeons who believed that there was always a relationship had the following profile: < 5 years of experience (41%), 22–50 primary TKA procedures per year (34%), no revisions (34%), and conducting 50% of their work on knees (Table 3). Knee surgeons who believed that TKA outcome was seldom related to psychiatric problems had the following characteristics: 15–30 years of experience (3%), > 100 primary TKA procedures per year (4%), > 5 revisions per year (3%), and conducting 50% of their work on knees (Table 4).

#### Psychiatric history

Only a few of the surgeons used a psychiatric evaluation questionnaire (3%) (Table 2), and only 20% always took a psychiatric history. Forty percent often took a psychiatric history, and 16% rarely or never used this kind of evaluation (Table 2).

Surgeons who always took a psychiatric history had the following characteristics: < 5 years of experience (28%), > 100 primary TKA procedures per year (25%), < 5 revisions per year (26%), and conducting 50% of their work on knees (22%). The differences were not statistically significant (Table 3). Surgeons who never or seldom took a psychiatric history belonged to the following groups: > 30 years of experience (18%), 50–100 primary TKA procedures per year (20%), > 5 revisions per year (20%), and specialising 100% in knees (17%) (Table 4).

#### Psychological or mechanical reasons for dissatisfaction

One third of the surgeons believed that psychological factors were a reason for patient dissatisfaction, a quarter believed that mechanical factors played the biggest role, and another quarter believed that combinations of factors were behind this (Table 5).

**Table 5:** TKA surgeon’s opinion about the general cause of dissatisfaction (*N* = 219)

Variables, *n* (%)	Surgeon’s opinion
Psychological/expectations	66 (30)
Mechanical/soft tissue	56 (26)
Combination	59 (27)
Unknown	14 (6)
Miscellaneous	8 (4)
Missing	16 (7)

The table shows what knee surgeons thought in general as the main cause of patient’s non-satisfaction after primary total knee arthroplasty

### Thematic analysis: Reasons for dissatisfaction after knee surgery

Five categories emerged in the thematic analysis, and are discussed below in descending order of their frequency of being mentioned.

#### Patient expectations

Almost half of the surgeons (122/262) stated that patients’ expectations were a predictive factor of bad outcome. They described these expectations as too great, unrealistic, high, wrong, and unreasonable.

#### Choice of patients to operate on

The second most common reason for dissatisfaction was the choice of patients to operate on. A considerable number of surgeons (72/262) related bad outcome to patient selection. Their statements included mentions of bad preoperative physical and psychological plan, inadequate motivation for postoperative training, multiple previous surgeries, surgery in early stages of arthrosis, mild complaint, obesity, low pain threshold, anxiety, depression, wrong indication, co-morbidities, poor preoperative range of motion, and insufficient rehabilitation.

#### Surgical factors

The third factor was related to surgery. Many of the orthopaedic surgeons (41/262) mentioned surgery-related factors as a cause of dissatisfaction after TKA, including unskilled/inexperienced surgeon, insufficient surgical performance, mechanical issues, poor implant positioning, no implant better than the natural knee, scar tissue, instability, swelling, soft tissue envelope, ligament balance, and patella.

#### Combination of factors

A few of the surgeons (18/262) stated that poor outcome was determined by a combination of factors. This was the fourth common cause mentioned by the surgeons.

#### Insufficient information

The fifth and least commonly-mentioned factor that determined the poor outcome was patient information. Very few of the surgeons (9/262) mentioned this as an important factor, and none named it as a sole factor; it was mainly grouped with patient expectations.

## Discussion

The Swedish orthopaedic surgeons in the present study described preoperative patient expectations as an important issue in predicting outcome after TKA surgery. However, this descriptive study revealed deficiencies among many TKA surgeons in supplying preoperative information. The discussion below aims to outline the extent of this problem among knee surgeons in Sweden.

All knee surgeons provided some kind of information to their patients preoperatively. However, only 58% of knee surgeons always provided written information, though 92% always informed their patients verbally. Preoperative information included information about indications, contraindications, surgical procedure, risks and benefits, outcome, and prognosis. Preoperative written and oral information has been shown to reduce postoperative pain and thereby enhance postoperative outcome [40, 41, 48], and a qualitative study on TKA patients showed that patients who were well informed preoperatively trusted their health care providers [40]. However, an earlier study showed that preoperative information about anatomy and patho-anatomy had a limited effect on pain management, while information about pain was more effective [42]. Furthermore, it is of utmost importance that the patients understand the information given. A recent study found that patients with low health literacy had impaired postoperative recovery and lower postoperative quality of life [49].

Written information in the form of booklets has been shown to have a positive effect on outcome after TKA [48]. Another study pointed out the importance of providing both verbal and written information together in order to facilitate postoperative pain control [41]. Consequently, the preoperative provision of written information to all TKA patients could offer a way to increase the satisfaction rate.

In terms of asking about patients’ expectations, only 44% of the surgeons always discussed this important issue with their patients. An earlier study showed that TKA surgery failed to meet the patients’ expectations when it came to kneeling, squatting, and stair climbing, and in particular that the fulfilment of expectations was highly correlated with satisfaction [43]. Tilbury et al. came to a similar conclusion, emphasizing the importance of preoperative information and education due to the substantial number of TKA patients with unfulfilled expectations [44]. It is very important for the surgeon to ask about the patient’s expectations, and make it clear to them which activities might be difficult to perform after the surgery. A study revealed that only young, strong patients who did not have a problem with ascending or descending stairs preoperatively were likely to be able to use stairs postoperatively without a problem [32]. Expectations of improvement in this functional ability may thus contribute to patients’ feeling disappointed after surgery, and so impelling knee surgeons to ask their patients about expectations may decrease the rate of dissatisfaction due to unfulfilled expectations. Patient’s expectations on the outcome of the TKA are not only based on the information given by the knee surgeons, but rather from discussions with other people like friends, family and from information in media [50].

It is not known whether information about dissatisfaction rate affects outcome after TKA. Earlier research revealed that up to 20% of TKA patients were disappointed with their results [3, 4, 19, 22] .We therefore suggest that informing patients about the dissatisfaction rate before surgery is of importance, as it could increase their awareness of the expected success rate. Further research is needed to create an individualized risk prediction’s tool.

Only 42% of surgeons in our study always discussed the success rate after TKA surgery, and 10% never discussed this with their patients. Previous studies have shown that the severity of osteoarthritis correlates with satisfaction rate. Schnurr et al. found that patients suffering from mild or moderate osteoarthritis were at risk of dissatisfaction after TKA, and recommended that patients should be told about this [45]. Another study showed a similarly high dissatisfaction rate among people with mild osteoarthritis changes, and also revealed a high prevalence of chronic non-orthopaedic conditions among these patients, including anxiety/depression, fibromyalgia, low back pain, and prior brain injury [13].

Our Swedish data showed a high percentage (31%; 68/219) of knee surgeons who sometimes, often or always operated on painful knees with mild radiological osteoarthritis in patients with anxiety/depression. Previous medical history is fundamental in preoperative evaluation. Nonetheless, medical and psychiatric illnesses are equally important, and should always be included in preoperative judgment. In recent years, there has been more recognition of the impact of psychological factors on joint prosthesis outcome. Many studies show a negative relationship between depression/anxiety and prosthesis outcome [3, 4, 6, 9, 12, 13, 16, 17]. However, only 20% of the surgeons in our study always took a psychiatric history, and 10% never or rarely enquired about psychiatric problems. Earlier research has shown that the rate of depression is 10–13% among the arthroplasty population, and that depression is correlated with an increased risk of poor outcome after surgery [8, 9]. Thus, awareness of this patient category needs to be increased, at least among Swedish knee surgeons.

Many studies recommend preoperative evaluation and management of psychiatric problems to mitigate postoperative complaints, thereby decreasing dissatisfaction rate after TKA [3, 8, 11, 15, 17, 18, 24, 51, 52]. In our survey, only 10% of orthopaedic surgeons always consulted a psychiatrist when they suspected a psychiatric problem, and 16% never or rarely did this. Moreover, only 3% of the surgeons used preoperative psychiatric questionnaires. With the support of the above-mentioned literature, the use of this kind of questionnaire can detect patients with a psychiatric disorder. There are no systematic protocols yet in Sweden to refer patients with psychiatric diseases for a professional psychiatric evaluation before TKA surgery. We recommend to build up such systems which we consider as important as referral for physical evaluation before surgery.

The knee surgeon’s responses were consistent with literature considering their believes about the causes of dissatisfaction. This was regarded patients’ expectations [12, 20, 32, 43, 44, 53], the choice of patient to operate on [3, 7, 13, 15, 18, 45, 52, 54–56], surgery related factors [21, 57, 58], combination of factors [10] and poor provision of information [12, 40, 44, 48, 59].

One limitation of this study is its descriptive design; However, the study does reveal the extent of the knee surgeons who do not provide a sufficient preoperative patient information in Sweden, which may be similar in other countries. Descriptive studies are ranked low in the hierarchy of evidence [60], but the strength of this survey is that it is unique in describing for the first time Swedish knee surgeons’ attitude to preoperative information. In addition, the qualitative analysis of surgeons’ beliefs shows how surgeons think about dissatisfaction after TKA patients in Sweden. Another weakness is the absence of psychometric analysis of the questionnaire. The questionnaire was constructed by the researchers with the aim of investigating the attitudes of knee surgeons, as there was no validated questionnaire which could answer the aim of the study. Many differences between the groups were statistically non-significant because of multi-categorical comparison between the groups. When we condensed these categories, the differences became statistically significant, however without clinical importance. The high response rate added more strength to this study.

Another limitation of the study is that it only evaluated frequency of information provision, which does not tell us anything about the quality of this information, which may also be important and influence patients’ expectations and satisfaction [35, 40–42, 48, 59, 61]. Moreover, the surgeon’s believes and attitude that showed by the study may not represent the actual daily behavior of knee surgeons.

## Conclusions

The findings in this survey show that Swedish knee surgeons are aware of factors which predict poor outcome after knee arthroplasty surgery, and that they take these factors into consideration when verbally informing patients preoperatively. On the other hand, more effort is needed in improving written information, analysis of preoperative patient expectations, taking a psychiatric history, consulting a psychiatrist, and not operating on mild indications. Many studies support the negative effect of these factors on outcome. Changing the attitudes of knee surgeons towards preoperative care might decrease dissatisfaction rate. This survey may serve as background for future research regarding preoperative patient selection, surgeon attitudes, and satisfaction rate.

## Appendix 1

**Table 6:** Swedish hospitals which participated in the survey. SPSS statistical program

No.	Hospital	Knee surgeons (total no.)	percent	Knee surgeons (answered)
1.	Akademiska Uppsala	8	2.8	2
2.	Aleris Motala	4	1.4	3
3.	Alingsås	5	1.7	4
4.	Blekinge	7	2.4	7
5.	Bollnäs	3	1.0	2
6.	Capio Movement/Halms.	5	1.7	2
7.	Carlanderska	2	0.7	2
8.	Danderyd	6	2.1	6
9.	Eksjö-Nässjö-Högla	4	1.4	4
10.	Elisabeth/Uppsala	1	0.3	1
11.	Enköping	6	2.1	6
12.	Falun	6	2.1	2
13.	Gävle	6	2.1	6
14.	Halmstad	7	2.4	3
15.	Helsingborg	5	1.7	1
16.	Huddinge	7	2.4	1
17.	Hudiksvall	3	1.0	1
18.	Hässleholm	3	1.0	2
19.	Kalmar	3	1.0	3
20.	Karlskoga	5	1.7	5
21.	Karlstad	5	1.7	3
22.	Karolinska /Solna	4	1.4	4
23.	Kungsälv	4	1.4	4
24.	Lidköping	5	1.7	3
25.	Lindesberg	6	2.1	6
26.	Ljungby	5	1.7	3
27.	Lycksele	2	0.7	2
28.	Mora	6	2.1	1
29.	Mälar/Eskilstuna	8	2.8	7
30.	Nacka Aleris	3	1,0	3
31.	Norrtälje	3	1.0	3
32.	Nyköping	4	1.4	2
33.	Orthocenter/Göteborg	3	1.0	3
34.	Orthocenter/Löwenstr	4	1.4	4
35.	Ortopediska huset	4	1.4	3
36.	Oskarshamn	3	1.0	3
37.	Piteå/Sunderby	6	2.1	5
38.	Ryhov/Jönköping	3	1.0	1
39.	Sahlgrenska/Mölndal	12	4.2	10
40.	Skellefteå	4	1.4	4
41.	Skene/Södra Älvsborgs	5	1.7	5
42.	Skövde	3	1.0	3
43.	Sollefteå	2	0.7	2
44.	St. Görans	4	1.4	4
45.	Sundsvall	2	0.7	2
46.	SUS(Lund/Malmö)	16	5.6	10
47.	Södertälje	5	1.7	5
48.	Södersjukhuset	9	3.1	8
49.	Torsby	4	1.4	2
50.	Uddevalla	5	1.7	2
51.	USÖ/Örebro	3	1.0	3
52.	Varberg	5	1.7	5
53.	Vrinnevis/Norrköping	4	1.4	4
54.	Värnamo	4	1.4	4
55.	Västervik	3	1.0	3
56.	Västerås	5	1.7	2
57.	Växjö	7	2.4	7
58.	Ängelholm	3	1.0	3
59.	Örnsköldsvik	3	1.0	3
60.	Östersund	5	1.7	5
		287	100	219

The table shows the numbers and names of participating hospitals. It shows also the total number of knee surgeons who are working in each hospital and the number of knee surgeons who participated in the survey

## Appendix 2

**Table 7:** The questions answered by knee surgeons in the survey

No.	Question text
1	Do you use **written** information with clear text about risks, complications and benefits of TKA surgery?
2	Do you use **oral** information with clear text about risks, complications and benefits of TKA surgery?
3	How many years do you work with Knee arthroplasty?
4	How many primary TKA do you operate per year?
5	How many knee revision arthroplasty do you operate per year?
6	The percentage of working with knee surgery: a. 100% knee specialist b. 75% c. 50% D. 25%
7	Do you ask patients about their **expectations**?
8	Do you inform patient that about 20% of patients are not satisfied after TKA surgery despite the absence of obvious explanation?
9	If you get a patient with severe knee pain, mild radiological arthrosis, large desire to be operated and the patient has anxiety/depression. How often do you proceed with surgery?
10	Do you enquire routinely about psychiatric history?
11	If you realize that patient suffering from depression/anxiety, do you consult psychiatrist before proceeding with surgery?
12	Do you use a psychiatric enquiry sheet for evaluation of psychiatric problems?
13	If you use a psychiatric enquiry which reveal depression or anxiety, do you control that patient received firstly psychiatric treatment
14	Do you think that psychiatric problem play some role in the results?
15	Many of dissatisfied patients describe a pain. What do you think generally the most important reason to the pain if we excluded radiating pain
16	What do you think about the single most important reason that patient is dissatisfied after TKA

The table shows the English translated version of the questionnaire which was sent to the Swedish knee surgeons

## Abbreviations

SPSS: Statistical Package for the Social Sciences
TKA: Total knee arthroplasty
